# A novel DNA methylation score accurately predicts death from prostate cancer in men with low to intermediate clinical risk factors

**DOI:** 10.18632/oncotarget.12377

**Published:** 2016-09-30

**Authors:** Amar S. Ahmad, Nataša Vasiljević, Paul Carter, Daniel M Berney, Henrik Møller, Christopher S. Foster, Jack Cuzick, Attila T. Lorincz

**Affiliations:** ^1^ Centre for Cancer Prevention, Wolfson Institute of Preventive Medicine, Barts and The London School of Medicine, Queen Mary University of London, London, EC1M 6BQ, UK; ^2^ Centre for Molecular Pathology, Royal Marsden Hospital, Sutton, SM2 5PT, UK; ^3^ Molecular Oncology Centre, Barts Cancer Institute, Queen Mary University of London, London, EC1M 6BQ, UK; ^4^ King's College London, Cancer Epidemiology and Population Global Health Program, London, SE1 3QD, UK; ^5^ HCA International, Pathology Laboratories, London, WC1E 6JA, UK

**Keywords:** prostate cancer, progression biomarkers, DNA methylation, CAPRA score, survival analysis

## Abstract

Clinically aggressive disease behavior is difficult to predict in men with low to intermediate clinical risk prostate cancer and methylation biomarkers may be a valuable adjunct for assessing the management of these patients. We set to evaluate the utility of DNA methylation to identify high risk disease in men currently considered as low or intermediate risk. DNA was extracted from formalin-fixed paraffin-embedded transurethral prostate resection tissues collected during the years 1990−96 in a watchful-waiting cohort of men in the UK. The primary end point was death of prostate cancer, assessed by reviewing cancer registry records from 2009. Methylation was quantified by pyrosequencing assays for six genes (*HSPB1, CCND2, TIG1, DPYS, PITX2,* and *MAL*) with established biomarker value in prostate cancer. A novel prognostic methylation score was developed by multivariate Cox modelling using the six methylation biomarkers in 385 men with low-and-intermediate clinical risk variables and its prognostic value compared to two previously defined clinically-derived risk scores. Methylation score was the most significant variable in univariate and bivariate analysis in men with low-to-intermediate CAPRA risk score. When combined with CAPRA score the hazard ratio was 2.02; 95% confidence interval, 1.40−2.92. For a methylation score sensitivity of 83% the specificity was 44%, while the maximum achieved sensitivity by CAPRA was 68% at a specificity of 44%. The derived methylation score is a strong predictor of aggressive prostate cancer that could have an important role in directing the management of patients with low-to-intermediate risk disease. The estimated areas under the curve (AUC) at 10 years of follow-up were 0.62 (95% CI: 0.51, 0.70) and 0.74 (95% CI: 0.65, 0.82) for CAPRA, and combined (CAPRA + methylation) risk score (CRS) respectively.

## INTRODUCTION

Current inability to distinguish biologically indolent prostate cancer from that which will progress poses the greatest problem when deciding appropriate clinical management strategies for this disease. Although diagnosis by PSA may offer better outcomes by early treatment, [1] lack of specificity encourages overtreatment of indolent disease [2]. Following diagnosis, prostate cancer patients are commonly categorized as low, intermediate, and high risk to aid management decisions. Tools such as CAPRA (Cancer of the Prostate Risk Assessment) score, [3] which combines baseline PSA level, Gleason score, age, and other clinical variables can provide some qualitative measure of a patient's risk for progression. However, three of the strongest weighting variables in defining clinical scores, i.e. T-stage, biopsy Gleason score, and malignancy ratio of the biopsy cores, are subjective, and prone to interpretational bias, and can dramatically overestimate or underestimate the actual risk classification in any given patient. The prognostic value of TMPRSS2:ERG, [4] oncotype DX prostate cancer assay, [5] four kallikreins panel [6] and cell cycle progression (CCP) score [7] have so far shown varying potential utility. However, a pressing need still exists to validate additional scores for clinical use as well as identify standardized quantifiable molecular biomarker assays, or optimal combinations of biomarkers to improve disease stratification and subsequent management.

DNA methylation exists in the human genome in complex patterns and is essential for normal development in higher organisms. Deregulation of methylation is a common event in carcinogenesis with abnormal methylation contributing to both the occurrence and progression of prostate cancer [8–10]. In a recent report, we demonstrated that changes in methylation of *HSPB1, CCND2*, *TIG1, DPYS*, and, *MAL,* had significant prognostic effects in multivariate Cox models where death from prostate cancer was the study endpoint [11]. In an additional matched case-control study using a subset of patients with low and intermediate Gleason score, methylation of the *PITX2* gene was informative for identification of men at high risk of aggressive prostate cancer [12]. Here we propose a new risk stratification score utilizing the methylation levels of the six genes: *HSPB1, CCND2, TIG1, DPYS, PITX2,* and *MAL,* that enhance identification of men with aggressive cancer who would otherwise be considered of low or intermediate risk based on clinical variables. The new classifier allows a more robust classification to segregate men of low risk who can be safely followed by active surveillance from those who require active intervention.

## RESULTS

Of 573 eligible patients, 385 men were grouped into low-and-intermediate-risk CAPRA scores, of which 57 (14.8%) died from prostate cancer, 188 (48.8%) died of other causes, and 140 (36.4%) were alive at last follow-up (December 2009) (Supplementary Figure S1). The distribution of the candidate predictors is shown in Supplementary Table S1.

Schoenfeld residuals demonstrated no violation of the assumption of proportional hazards in any tested variable, therefore a multivariate Cox proportional hazards model was fitted with the six genes in the 385 men with low-intermediate-risk CAPRA scores. A DNA methylation score was developed from the multivariate Cox model (Supplementary Figure S2), which has the following form:

Methylation score = 0.543*log(1 + *HSPB1*) + 0.357*log(1 + *CCND2*) − 0.349*log(1 + CCND2*HSPB1) + 0.354*log(1 + *TIG1*) + 0.230*log(1 + *DPYS*) + 0.182*log(1 + *PITX2*) + 0.118*log(1 + *MAL*).

The interaction term of CCND2*HSPB1 was added to the model because combined methylation of both genes together was negatively associated with death from prostate cancer (Supplementary Figures S3 and S4).

Univariately, the methylation score was the strongest predictor of prostate cancer related death with a hazard ratio [HR] 2.72, *p* < 10^−8^ compared to the CAPRA score HR 1.62, *p* < 10^−7^ (Table 1). Also in a bivariate analysis, the methylation score was the strongest predictor with HR: 2.02, *p* < 10^−3^. No significant interaction was observed between methylation score, and CAPRA score. The methylation score showed a weak correlation to extent of disease (Spearman's rho = 0.39) and CAPRA score (Spearman's rho = 0.38) (Supplementary Figure S5). The associated *p*-values were 3.2e-15 and 5.2e-15 respectively.

**Table 1:** Univariate analysis for the methylation score and the CAPRA score (linear) as well as bivariate Cox analyses with the methylation score, and the CAPRA score (linear) as predictors

	Univariate		Bivariate model	
marker	HR (95% CI)	Χ_1_^2^ (p)	HR (95% CI)	Χ_1_^2^ (p)
Methylation	2.72 (1.93, 3.83)	34.54 (4.2e–09)	2.02 (1.40, 2.92)	13.91 (1.9e–04)
CAPRA	1.62 (1.36, 1.91)	31.05 (2.5e–08)	1.40 (1.15, 1.69)	11.86 (5.7e–04)
Χ^2^ (p)			46.26 (9.0e–11)	
c-index (se)			0.74 (0.04)	

In a sensitivity analysis, using Jackknife (leave-one-out) resampling, the alternatively developed score using parameterwise shrinkage factors for Cox regression confirmed the strong performance of methylation score (Supplementary Table S2). A second sensitivity analysis, where an alternative imputation method was used to predict the probability of T-stage III values (Supplementary Figure S6) further confirmed that methylation score had strong prognostic value (Supplementary Table S3). A log-rank chi-square test was used to compare survival curves of three subsets; omitted samples (*n* = 146; death from prostate cancer = 36); samples without missing values (*n* = 333, death from prostate cancer = 94), and samples with missing T-stage (*n* = 240, death from prostate cancer = 67). The Kaplan-Meier curves of the three groups overlap (data not shown), suggesting that there is no subset difference in survival (log-rank chi-square test = 1.16, d.f. = 2, and *p* = 0.56).

**Figure 1:**
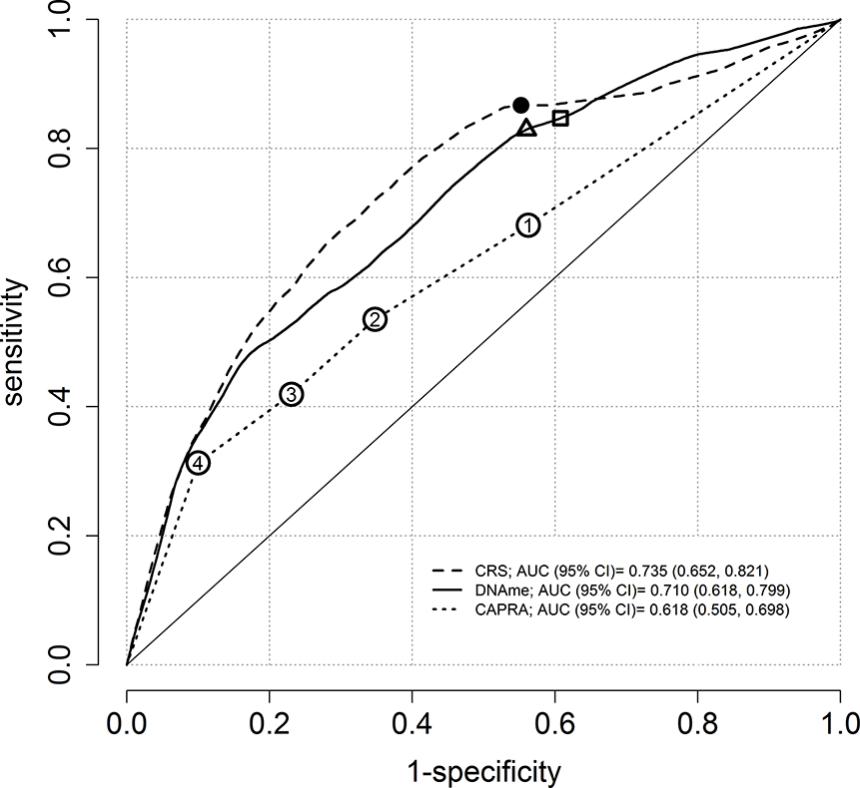
Time-dependent ROC curves at ten-years of follow-up using the semiparametric monotone sequence efficient estimator for three prostate cancer risk scores For the methylation (DNAme) score, 85% sensitivity and 39% specificity is indicated by a square. Sensitivities and specificities for CAPRA categories 1, 2, 3 and 4 are indicated with circles, where 1 achieved the maximum possible sensitivity of 68% and 44% specificity. If equalized to obtain the same specificity, the methylation score displayed sensitivity of 83% (triangle). The solid circle presents the optimum reached sensitivity of 87% and specificity of 45% for the combined risk score (CRS). The confidence intervals of AUC are based on 1000 bootstrap replicates.

The estimated areas under the curve (AUC) at 10 years of follow-up were 0.62 (95% CI: 0.51, 0.70), 0.71 (95% CI: 0.62, 0.80), and 0.74 (95% CI: 0.65, 0.82) for CAPRA, methylation, and combined (CAPRA + methylation) risk score (CRS) respectively (Figure 1). A bootstrap test with B = 1000 was performed to compare the AUCs of the CAPRA score and the CRS [13]. A statistically significant difference was observed between the AUC of CAPRA and CRS (*p* = 0.01). The optimum cut-off value for the methylation score was 2.34 and yielded 85% sensitivity and 39% specificity while the optimum cut-off CAPRA = 1, reached 68% sensitivity and 44% specificity. In comparison, at a cut-off (2.43) where methylation score reached the same specificity, a sensitivity of 83% was observed (Figure 1).

**Figure 2:**
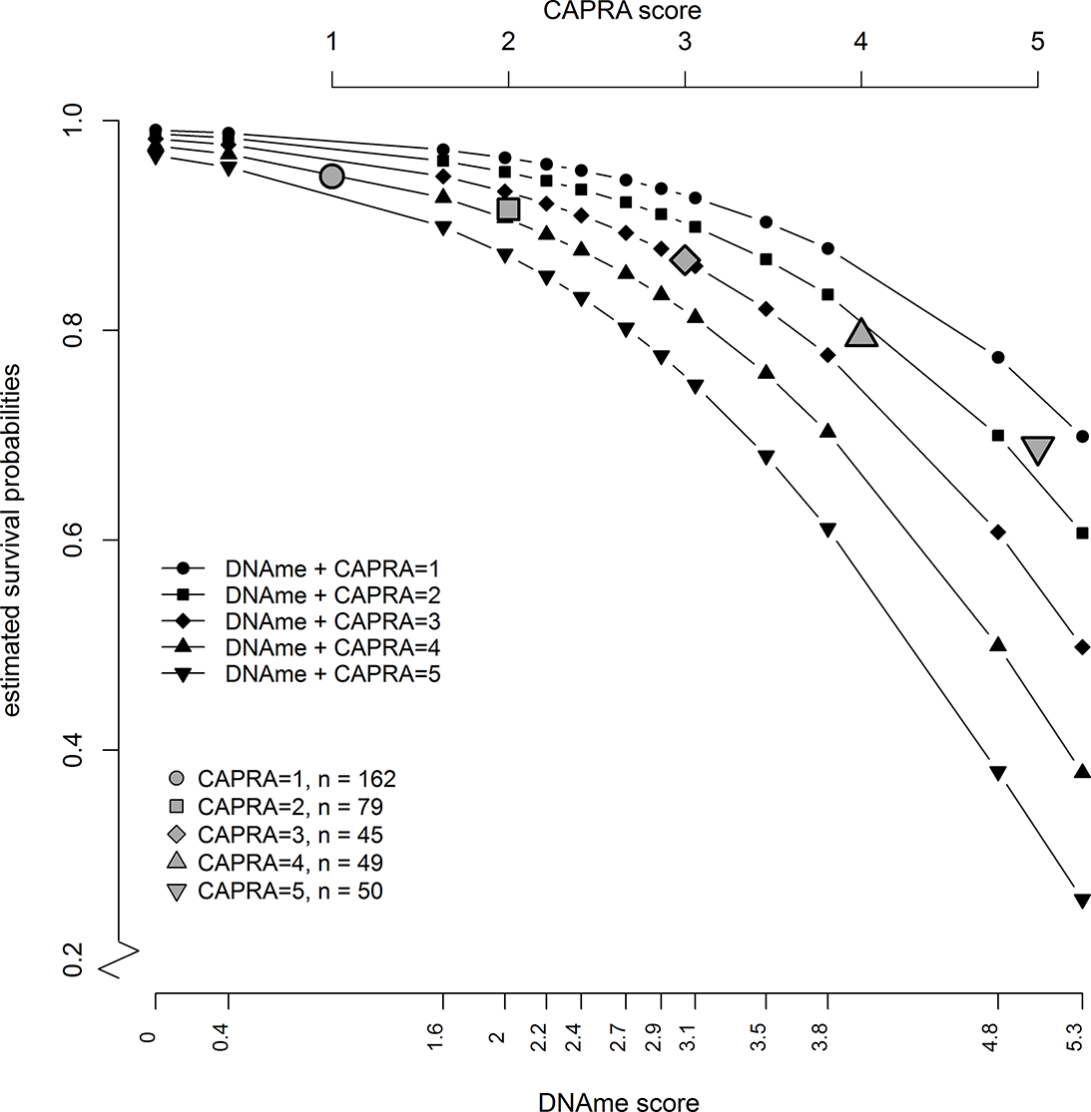
Estimated absolute risk values from a Cox model with combined risk score as predictors (black symbols) show that a patient with any given CAPRA score would be likely to get a more accurate risk stratification depending on the level of methylation (DNAme) of the six genes in the methylation score The gray symbols show the estimated survival probabilities from a univariate Cox model if CAPRA score alone is used as a predictor.

Figure 2 presents the estimated absolute risk values from a Cox model with methylation score, and CAPRA score as predictors showing that the survival probabilities in all CAPRA groupings (CAPRA = 1–5) decrease as methylation score increases. Thus, rather than discrete survival probabilities based on CAPRA alone, introducing the CRS allows a further prediction of death with methylation percentile. The Harrell c-index indicates a good discriminatory capacity of predictive performance of the methylation score (Table 2). We also performed a competing risks analysis using the Fine-Gray regression model [14, 15] for the cumulative incidences of the competing events, death from prostate cancer, death from other causes, and men still alive at censoring. We performed univariate, and bivariate analysis (Supplementary Table S4); the risk factors investigated were methylation -score and CAPRA score. Similar to the main analysis, the methylation score was the strongest independent predictor of death from prostate cancer in univariate and bivariate competing risk analyses.

**Table 2:** The predictive performance of methylation with respect to calibration and discrimination, using 5-fold cross-validation (repeated 100 times and averaged)

	Calibration Slope	Harrell's c-index
estimate (95% CI)	0.828 (0.604, 1.063)	0.678 (0.647, 0.708)

The cross-validated calibration slope (slope of predicted log hazard vs. true log hazard) was 0.83, indicating acceptable fit. The Harrell c-index was 0.68, indicating good discriminatory capacity. This c-index value of 0.68 from the internal validation is an honest estimate of internal validity, which penalizes for overfitting. The above table shows the average of 100 repeated values of the estimated calibration slope and estimated Harrell's c-index with (α/2; 1−α/2) percentile confidence interval, where α = 0.05.

## DISCUSSION

This is the first study to establish the prognostic value of a DNA methylation score as a test for men with low-and-intermediate risk CAPRA-score prostate cancer which can be further combined with CAPRA into a powerful combined risk score. Our findings support the original hypothesis that methylation status of six selected index genes (*HSPB1, CCND2, TIG1, DPYS, PITX2,* and *MAL*) provides a novel, objective, and accurate prediction of death from prostate cancer in men clinically assessed as low to intermediate risk. The biomarkers in the classifier have different coefficients, consistent with varying individual effect sizes. *HSPB1* and *CCND2* are the two strongest and most predictive biomarkers and in combination with the other four genes gave a substantially higher sensitivity for aggressive behavior than was possible using CAPRA whatever threshold was used.

The methylation score was compared to the CAPRA score, comprising well-known clinical parameters: PSA level, Gleason score, T-stage, percentage of positive biopsy cores and age of the patient at diagnosis. Methylation score was the strongest predictor of prostate cancer related death univariately as well as in bivariate analyses, although both elements contributed additional independent information to the combined risk score. This suggests that measurements of methylation score and CAPRA represent independent aspects of disease aggressiveness and both are required to improve the accuracy of prognosis. Therefore, patients managed by watchful-waiting or active surveillance can have the benefit of significant pre-treatment prognostic information from the methylation score.

The novelty of our work is underpinned by accurate measurement of methylation in a common set of well-annotated clinical specimens from a large retrospective cohort with long follow-up. Furthermore, the risk of overfitting was minimized by keeping the gene panel small, thus reducing problems that often occur in studies with microarrays and deep sequencing of thousands of genes in small sample sets. The large size of Transatlantic Prostate Group (TAPG) cohort and a follow-up exceeding 10 years are two major strengths of our study. The TAPG cohort provides information on death from prostate cancer in a wide population from a country where PSA surveillance was available in a developed health service. This supports our ability to identify patients who would do well or poorly long-term with conservative management in a broader context than would be possible with many contemporary cohorts that use biochemical recurrence as this is a weaker surrogate end point. Our results suggest that the methylation score could accurately predict prostate cancer related death with a Harrell's c-index of 0.72, generally interpreted in the same way as an AUC, and it is plausible that extended studies may reveal additional methylation biomarkers to improve the overall performance.

Our methylation score is an objective multi- dimensional molecular classifier and is clearly distinct from a surrogate vectorial clinical classifier such as CAPRA score. It is also quite distinct from other molecular assessments such as the CCP score or Oncotype DX Prostate cancer assay [5, 7]. The established activities of *DYPS, MAL, and TIG1* are consistent with tumor suppression [16–18] while *CCND2, HSPB1 and PITX2* are mainly oncogenic [19–21]. In prostate cancer, *PITX2* was earlier shown to be a prognostic biomarker of biochemical recurrence in which increased methylation was associated with reduced mRNA transcription [22, 23]. The DNA methylation classifier assesses aspects of epigenetic modification which may determine the behavioral phenotype of an individual man with prostate cancer. Primary prostate cancers occurring in individuals are typically genetically heterogeneous [24], so that the efficacy of the new classifier depends upon the biological relevance of the individual focus of cancer biopsied. While other reports have identified hypermethylation as a powerful modality with which to confirm the presence of prostate cancer [25], only the new classifier has the apparent ability to accurately predict which men have poor outcome within the group of low-to-intermediate clinical risk.

Although in current practice, TURP diagnosed cancers usually are small and confined to the transition zone, our TAPG TURP cohort contained a greater percentage of aggressive cancers due to the particular practice patterns of the 1990. Hence, it does not reflect the finer points of contemporary practice in the diagnosis or treatment of prostate cancer. However, objective evidence shows that the TAPG cohort is well suited to both biomarker discovery and validation e.g. the same set of specimens was used to validate the CCP score that has now become commercially available as a routine clinical test [7, 26]. Another limitation of our study is that death from prostate cancer was relatively rare in this clinically low risk group of men and larger cohorts are needed to fully characterize the value of the methylation score in identifying higher risk patients. Validation of the methylation score in contemporaneous cohorts diagnosed by use of needle biopsy is an obvious next step.

In conclusion we have shown that a novel DNA methylation classifier provides additional prognostic information both for men with localized prostate cancer and also for their managing clinicians who face difficult choices between active surveillance versus immediate and potentially aggressive therapy. If generally confirmed in other settings, the methylation score is likely to make a major contribution to the management of prostate cancer worldwide. Further validation studies are needed, especially for screen-detected cancers that are diagnosed by needle biopsy in situations where active surveillance without initial treatment is an ethical option. The new DNA methylation score can aid clinical decision making in patients with prostate cancer who with current risk assessment methods are misidentified as low or intermediate risk.

## MATERIALS AND METHODS

### Patients

The study cohort includes well-characterized men residing in Great Britain who did not receive any treatment for minimum six months following diagnosis of prostate cancer. Patients were excluded if they were treated by radical prostatectomy, hormones, radio- or chemotherapy, showed objective evidence of metastatic disease, had a PSA measurement above 100 ng/mL or died at or within six months of diagnosis. Original histological specimens from the diagnostic procedure were requested, collected, and diagnosis centrally reviewed by a panel of expert urological pathologists. Gleason scores were reassigned by use of a contemporary interpretation of the scoring system [27].

National ethical approval was obtained from the Northern Multicenter Research Ethics Committee followed by local ethics committee approvals from each of the collaborating hospital trusts [28].

### Specimen characteristics

719 formalin-fixed paraffin wax-embedded (FFPE) transurethral resection of prostate (TURP) tissues from the TAPG cohort were included (Supplementary Figure S1, and Table 3). The material was used as described in detail in a previous study [28].

**Table 3:** Overview of clinical and methylation variables in the entire TAPG patients (*N* = 719) and analyzed samples (*n* = 385)

variable name	variable	CAPRA points	*N* (%)	*n* (%)
Gleason Score	no pattern 4 or 5	0	375 (52.2%)	281 (73.0%)
	secondary pattern 4 or 5	1	123 (17.1%)	65 (16.9%)
	primary pattern 4 or 5	3	221 (30.7%)	39 (10.1%)
Clinical stage	T1 or T2	0	356 (49.51%)	376 (97.7%)
	T3	1	78 (10.85%)	9 (2.3%)
	NA	NA	285 (39.64%)	
PSA score (ng/mL)	PSA ≤ 6	0	315 (43.8%)	244 (63.4%)
	6 < PSA ≤ 10	1	82 (11.4%)	60 (15.6%)
	10 < PSA ≤ 20	2	113 (15.7%)	54 (14.0%)
	20 < PSA ≤ 30	3	74 (10.3)	20 (5.2%)
	PSA > 30	4	116 (16.1%)	7 (1.8%)
	NA	NA	19 (2.6%)	
Cancer in biopsy (%)	Extent < 34%	0	461 (64.1%)	316 (82%)
	Extent ≥ 34%	1	255 (35.5%)	69 (18%)
	NA	NA	3 (0.4%)	
Age at diagnosis	Age < 50	0	2 (0.3%)	1 (0.3%)
	Age ≥ 50	1	717 (99.7%)	384 (99.7%)
Sample size			Total = 719	Analyzed = 385

CAPRA-score points are indicated for comparison.

### Assay methods

Methylation data from 367 patients was obtained from previous studies [11, 12]. Methylation measurements for *HSPB1, CCND2, TIG1, DPYS, PITX2,* and *MAL* were performed on bisulfite converted DNA from an additional 352 patients as previously described [11, 12].

The number of included patients from the first and second group were 218 (57%) and 167 (43%) respectively. We have performed a statistical analysis to test for batch effect by comparing the Kaplan-Meier curves of the two groups. The two groups KM curves overlap, suggesting no subset difference in survival (log-rank chi-square test = 1.7 and *p*-value = 0.195). Furthermore, a univariate Cox model was fitted with a binary variable (1st group and 2nd group) as predictor. The group variable was not statistically significant (LR test chi-square = 1.667, *p*-value = 0.197). This suggests no batch effect was observed in the analyzed data.

### Study design

Men were included if they had clinically localized disease diagnosed by TURP from 1990–1996 inclusive, and were younger than 76 years at the time of diagnosis. The study median follow-up time was 11.36 years with IQR 6.20–14.72.

The CAPRA-score for each subject was computed, and 188 patients with high risk CAPRA scores (> 5) were excluded from the analysis with the rationale that these high risk men are not candidates for active surveillance, and will receive definitive treatment regardless of any additional molecular genetic information (Supplementary Figure S1). One patient with CAPRA = 0 (age 49.95 years) was included as part of the CAPRA = 1 group. Study endpoints were compared for the CAPRA variables and for gene methylation measurements of the six genes. Sample size calculations were not done, this was a large study and we selected samples from all men available from the archives.

### Statistical analysis

The statistical methods were documented in a pre-specified statistical analysis plan and laboratory testing was blinded from the clinical variables to minimize bias in the results. Cox proportional hazards models were used to analyze the effects of covariates on the primary endpoint, death from prostate cancer. Patients were censored on the date of last follow-up, or death from other causes. Schoenfeld residuals were examined to determine if Cox modelling was an appropriate analytical approach. The Spearman's rank correlation was estimated between all variables and scores individually. Time-dependent receiver operator characteristic (ROC) curves were plotted at ten-year follow-up using the semiparametric monotone sequence estimator for the methylation score, CAPRA score, and the combined risk score (CRS). A relatively small span was chosen 0.25*n^−1/5^, where *n* = 385 is the number of observations, [29] which yields only moderate smoothing to facilitate comparison of the nearest neighbor estimator. Prostate cancer survival probabilities at ten-years were predicted from fitted univariate Cox proportional hazards models. Five-fold cross-validation was done using the R-validate command. This was repeated 100 times to obtain an average optimism, which was subtracted from the final model fit indexes (e.g. c-index, and calibration slope) to obtain the overfitting-corrected estimates [30]. In a first sensitivity analysis, a parameterwise shrinkage analysis for factors of regression coefficients from the multivariate fitted Cox regression with methylation of the six genes and the interaction between *CCND2* and *HSPB1* was performed using a Jackknife (i.e. leave-one-out resampling) analysis. An alternative methylation score was developed using the shrunken regression coefficients. In the second sensitivity analysis, the missing T-stage values were predicted by fitting a multivariate logistic regression with Gleason, PSA, log(1 + Extent), age at diagnosis as predictors, and T-stage as the response variable with T1&T2 (*n* = 270, 81%), and T3 (*n* = 63, 19%) in the 333 patients with data available for all clinical and methylation variables.

All applied statistical tests were two-sided, *p* < 0.05 were considered significant. No adjustment for multiple comparisons was made. Analyses were performed in R version 3.2.3.

## SUPPLEMENTARY MATERIALS FIGURES AND TABLES


