# Supplementary material for: A novel DNA methylation score accurately predicts death from prostate cancer in men with low to intermediate clinical risk factors
Source: Oncotarget. 2016 Sep 30;7(44):71833–40. doi: 10.18632/oncotarget.12377 (PMC5342126; doi:10.18632/oncotarget.12377)
Supplement: Supplementary file 1 [file oncotarget-07-71833-s001.pdf]

A novel DNA methylation score accurately predicts death from prostate cancer in men with low to intermediate clinical risk factors

Supplementary Materials

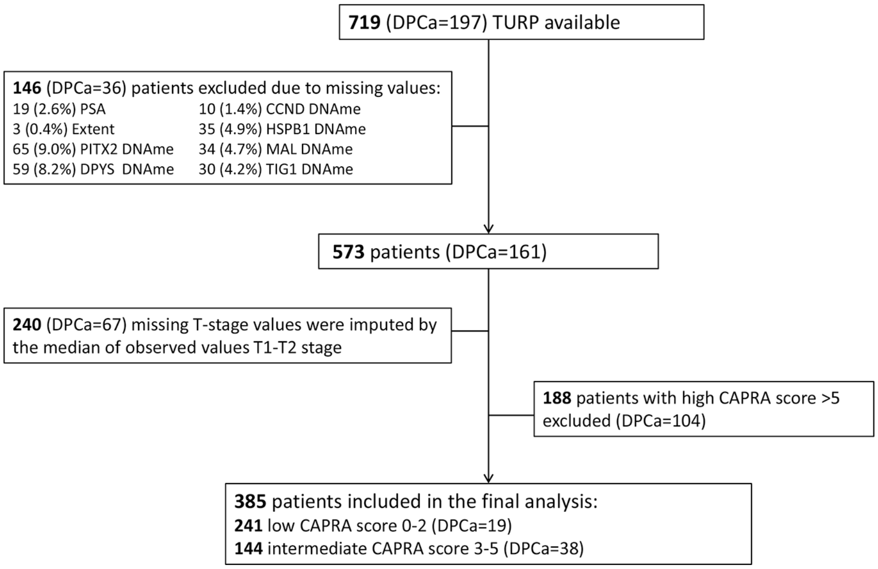

Supplemetary Figure S1: Samples with missing DNA methylation (DNAme) measurements are shown by the gene name and DNAme. DPCa=death from prostate cancer.

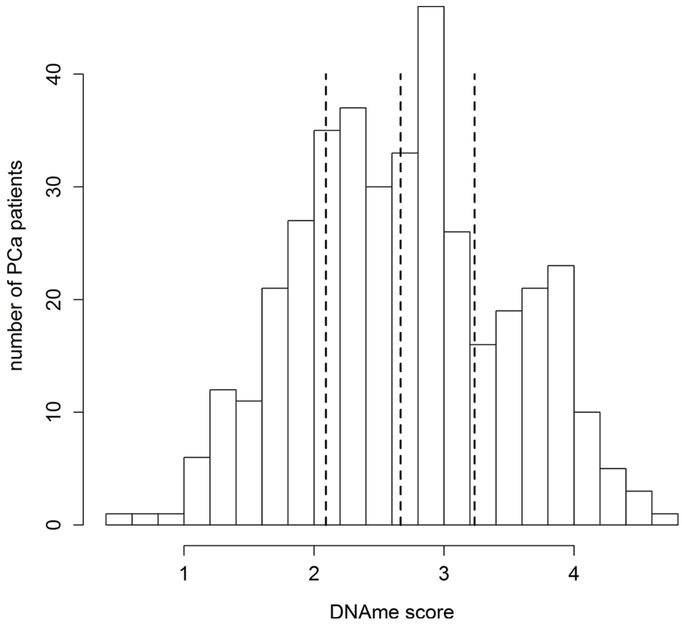

Supplemetary Figure S2: Distribution of the DNA methylation (DNAme) score in the dataset. The vertical dashed lines are the 25th= 2.1, 50th =2.7, and 75th =3.2 centiles of the DNAme score. PCa=prostate cancer.

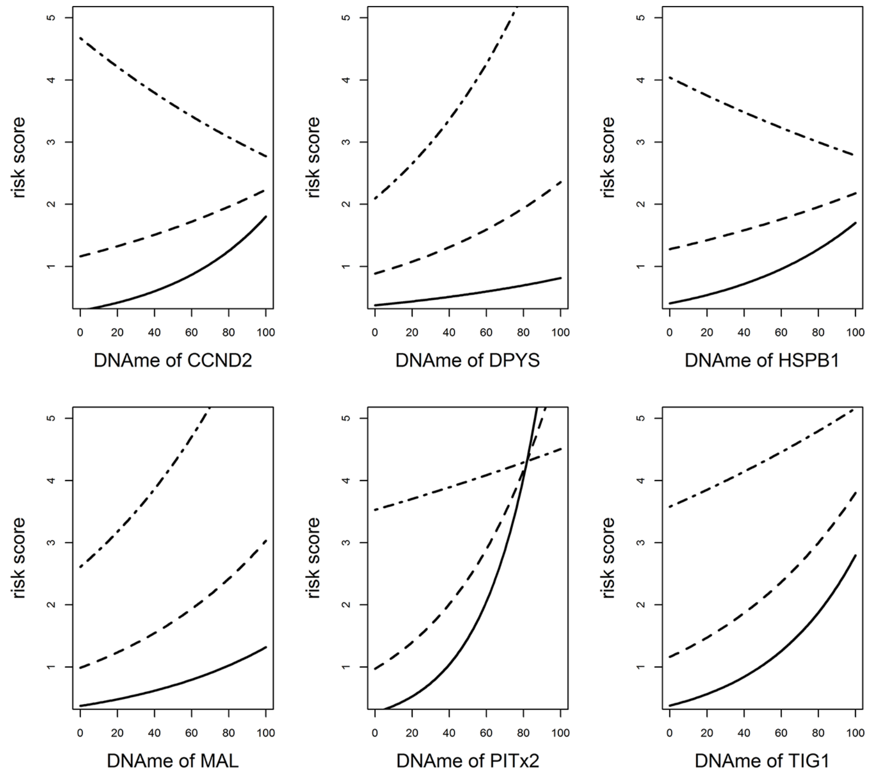

Supplemetary Figure S3: Predicted risk scores for the Gleason groups (Gleason<7 solid line, Gleason=7 dashed line, Gleason>7 dashed-dotted line) from a multivariate Cox model with Gleason + DNA methylation (DNAme) + Gleason*DNAme for each gene respectively. *CCND2* and *HSPB1* show statistically significant interaction with Gleason score, where patients with low Gleason become more at risk, when the DNAme of *CCND2* and *HSPB1* increases correspondingly.

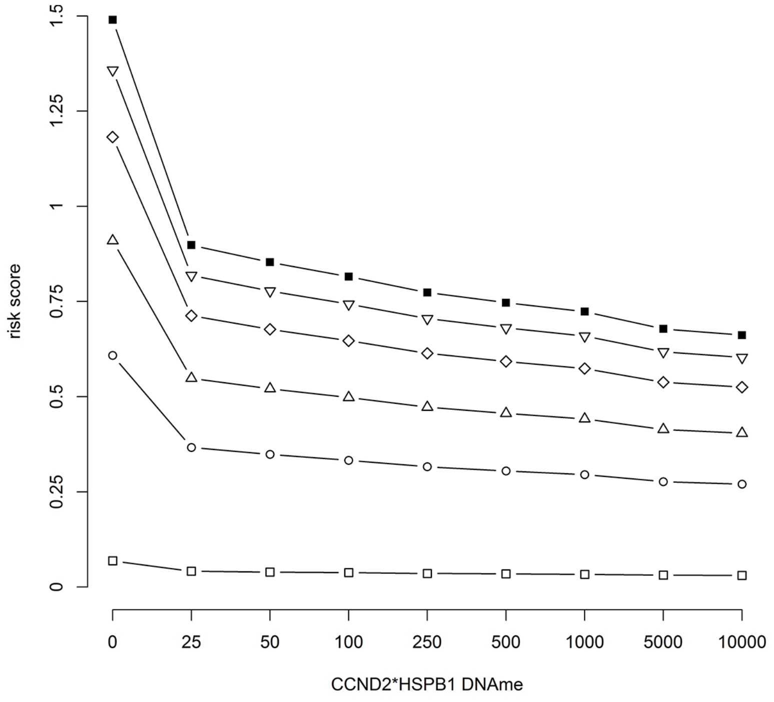

Supplemetary Figure S4: Predicted risk scores from a multivariate Cox model with CCND2 + HSPB1+ CCND2*HSPB1+DPYS+MAL+PITx2+TIG1 as predictors. The DNA methylation (DNAme) of the interaction term was varied from 0 to 10000 and risk scores were computed for constant DNAme of the other genes. The lines with squares, circles, triangles point-up, diamonds, triangles point down and filled squares points present the predicted risk score for constant 0%, 10% 25%, 50% 75% and 100% DNAme of the other genes respectively.

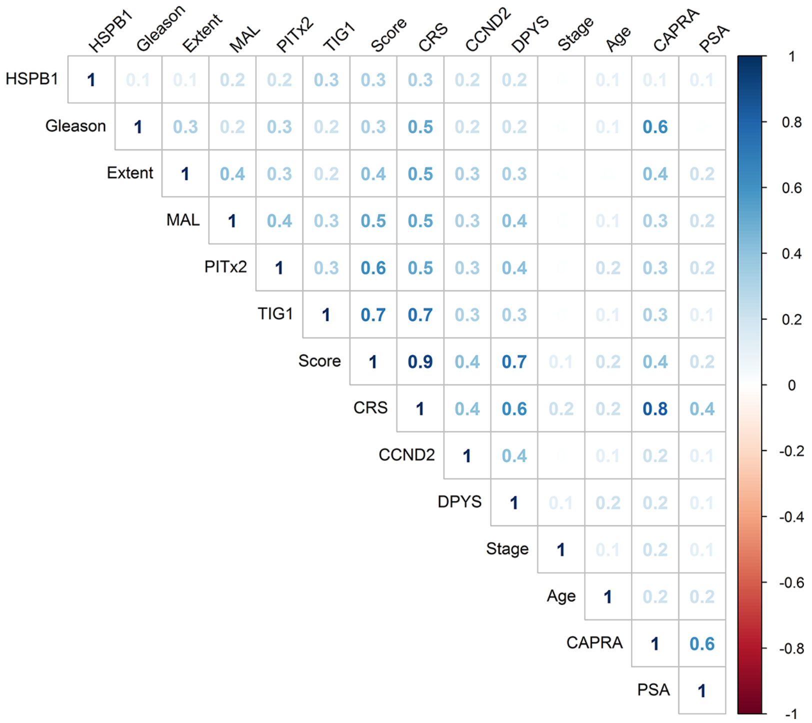

Supplemetary Figure S5: The estimated Spearman's rank correlation between all variables and scores.

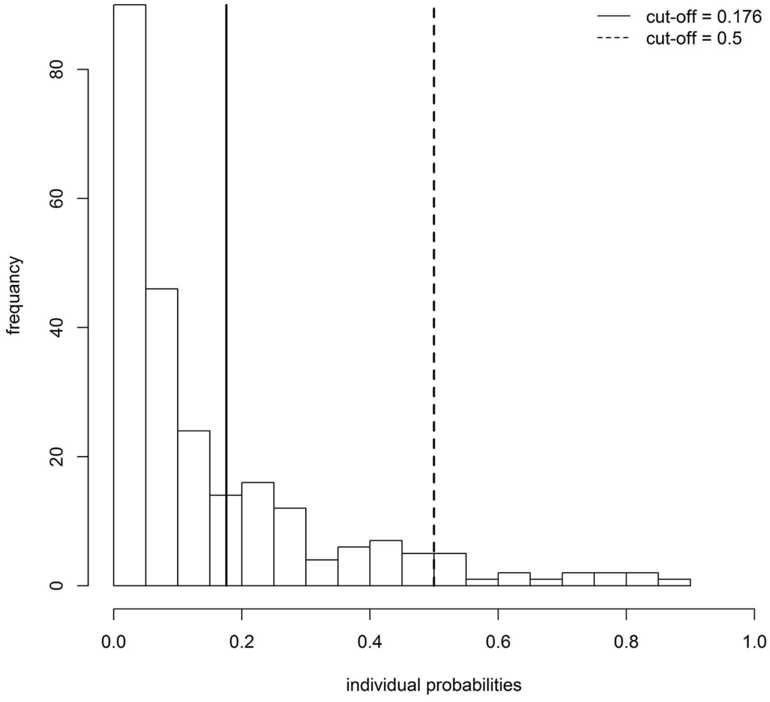

Supplemetary Figure S6: Predicted probabilities Y= Stage-III of the missing stage values (n=240, death from prostate cancer (DPCa=67) in the sensitivity analysis using Gleason, PSA, Extent and Age at diagnostic. The solid and dashed lines show the cutoff values of 0.176 and 0.5 respectively. These cutoff values were used to classify patients to Stage I and II (less than cutoff value) and Stage-III (greater or equal to cutoff value). The cutoff value of 0.176 gave highest Youden's J statistic in the test data (data without missing Stage, n=333, DPCa=94) and the decision rule's cutoff value of 0.5 (i.e. model correctly classified). Patients with missing clinical variables PSA and extent of disease, as well as invalid methylation measurements, were excluded from the statistical analysis (supplementary Figure S1). In addition, there were approximately 42% missing T-stage values. A categorical binary T-stage variable was recorded using 1 to indicate T3 samples and 0 for T1 or T2 samples. The missing T-stage values were imputed with the most frequent observed T1-T2 stage (47%) value.

Supplementary Table S1: Median, interquartile range (IQR) and number of missing values of each marker

marker	median (IQR: Q25-Q75%)	*N* missing values
Age	70.5 (6.3: 66.98–73.31)	0
PSA	8 (19.5: 2.5–22)	19
Extent (%)	16.7 (48.2: 4.85–53.0)	3
CCND2	30.1 (30.9: 14.70–45.60)	10
DPYS	35.6 (48.119: 10.96–59.08)	59
HSPB1	6.2 (16.81: 2.94–19.75)	35
MAL	21.3 (28.68: 9.92–38.60)	34
PITX2	18.6 (24.375: 8.1–32.48)	65
TIG1	5.3 (22.82: 2–24.817)	30

Supplementary Table S2: A sensitivity analysis of parameterwise shrinkage factors for the regression coefficients of the multivariate Cox model was performed by the Jackknife (i.e. leave-one-out resampling) method, considering methylation of the six genes and an interaction between *CCND2* and *HSPB1*

	Univariate			Bivariate		
	HR (95% CI)	LRT[a]	*p*	HR (95% CI)	LRT[a]	*p*
AltM	3.14 (2.06, 4.76)	30.58	3.20e–8	2.16 (1.39, 3.34)	11.87	0.001
CAPRA	4.21 (2.54, 6.99)	31.05	2.50e–8	1.43 (1.18, 1.72)	13.82	0.000
χ^2 (d.f., p)				44.26 (2, 2.45e–10)		
c-index (se)				0.733 (0.04)		

An alternative methylation score (AltM) was developed using the shrunken regression coefficients: AltM score= 0.026*log(1+*CCND2*)+0.112*log(1+*HSPB1*)-0.041*log(1+*CCND2*HSPB1*)+ 0.229*log(1+*DPYS*)-0.020*log(1+*MAL*)+0.088*log(1+*PITX2*)+0.378*log(1+*TIG1*).

[a] likelihood ratio chi-square test.

Supplementary Table S3: In a sensitivity analysis, the missing T-stage values were predicted by fitting a multivariate logistic regression with Gleason, PSA, log(1+Extent), age at diagnosis as predictors, and T-stage as the response variable with T1&T2 (*n*=270, 81%), and T3 (*n*=63, 19%) in the 333 patients with data available for all clinical and methylation variables

	T-stage cut-off = 0.176			T-stage cut-off = 0.5		
	***n* = 375, DPCa = 55**			***n* = 385, DPCa = 57**		
	HR (95% CI)	LRT[a]	*p*	HR (95% CI)	LRT[a]	*P*
DNAme	2.02 (1.41, 2.89)	14.77	0.0001	2.0 (1.37, 2.91)	12.87	0.0003
CAPRA	1.42 (1.17, 1.72)	12.89	0.0003	1.33 (1.12, 1.59)	10.39	0.001
χ^2 (d.f., p)	48.99 (2, 2.3e–11)			44.75 (2, 1.9e–10)		
c-index (se)	0.744 (0.041)			0.738 (0.040)		

All predictors were significant (p <0.05). The model likelihood ratio test was 80.72 (d.f.=4, p < 0.0001). AUC of the fitted multivariate logistic regression was 0.84 (95% CI: 0.78-0.89), which shows good utility in predicting the responses of individual subjects with Youden's index providing a sensitivity of 84% and specificity of 73%. The missing T-stage values of 240 men were imputed using predicted probabilities from this fitted logistic regression model choosing a cutoff value of 0.5 to best match the observed T-stage distribution. The resulting imputed T-stage values fell into the following groups: T1&T2 N = 224 (90%) and T3 N = 16 (10%). Bivariate analysis for death from prostate cancer in the sensitivity analysis with two cutoff values used for dichotomizing the predicted T-stage values. DNAme=DNA methylation.

[a] likelihood ratio chi-square.

Supplementary Table S4: Competing risks regression (Fine-Gray model) analysis of death from prostate cancer, Alive=140, DPCa = 57, and DOC=188

	univariate		bivariate		
	HR (95% CI)	PLRT[a]	HR (95% CI)	PLRT[a]	*p*
DNAme	2.56 (1.79, 3.66)	30.6	2.00 (1.41, 2.82)	15.36	8.90e-5
CAPRA	1.56 (1.32, 1.84)	26.1	1.36 (1.15, 1.61)	12.95	3.20e-4
pseudo LR test			41.1		

[a] Pseudo likelihood ratio chi-square test. DNAme = methylation score.
